# Revisiting the Causes of the Pull-to-Centre Effect: Evidence From China

**DOI:** 10.3389/fpsyg.2021.754626

**Published:** 2022-02-02

**Authors:** Lushuang Yang, Dahai Cai

**Affiliations:** Business School, Sichuan University, Chengdu, China

**Keywords:** ordering bias, Chinese newsvendor, overconfidence, cultural differences, monoculture

## Abstract

Prior experimental studies have shown that individuals' actual ordering decisions significantly deviate from the theoretical optimum in newsvendor problems and show the robust pull-to-centre (PTC) effect. Several human behaviours have been confirmed to be the causes of the PTC. However, most newsvendor experiments have been conducted in multicultural countries (e.g., the United States and Germany). As there exist mutual influences between culture and behaviour, in this study, we revisit the ordering biases in a monocultural country to examine the robustness of the PTC and whether the causes can still explain this phenomenon. Our results show that the PTC still prevails and heuristics still work. However, overconfidence cannot perfectly interpret the PTC in China for probable inconsistent confidence levels in individual judgments and decisions. Moreover, the “centre" may no longer be the mean demand but the average value of the realised demand. We explain these changes from the perspective of cultural differences. To be more specific, collectivism, holistic style, and *Doctrine of the Mean* play vital roles in Chinese newsvendors' decisions.

## 1. Introduction and Hypotheses Formulation

The premise of the classic newsvendor model is that decision makers are fully rational and pursue profit maximisation. However, recent experimental studies have challenged this premise by examining whether human decision makers expect profit maximisation, and seek the possible explanations for decision bias. Since the pull-to-centre (PTC) effect, that is “subjects order a quantity between the mean of the demand distribution and the expected profit-maximising quantity in the newsvendor setting,” was observed by Schweitzer and Cachon (2000), numerous studies have examined the robustness of this effect and found that the power of the PTC effect is dissimilar.

To explain the PTC effect, different theories have been tested, and several explanations (e.g., overconfidence, anchoring, and ex-post inventory error) have been proven to be valid. As a common nonstandard belief, overconfidence has been proven to be effective in explaining the PTC effect. Generally, overconfident (especially overprecise) individuals believe that their estimates are more accurate than they truly are (Moore and Healy, 2008). Ren and Croson (2013) explored the impact of overconfidence on the newsvendor's decision. In their setting, people underestimated the variance of the demand distribution and that leads to the PTC effect. Insufficient adjustment heuristics (Tversky and Kahneman, 1974), that is, people initially base decisions on an available anchor and then adjust towards the optimal decision based on further information, induce imperfect responses and suboptimal decisions. Two anchors in the newsvendor context are the mean demand and the previous ordering quantity. While the mean anchoring behaviour assumes that decision maker anchors on the mean demand and adjusts towards the optimal order quantity, the demand chasing behaviour assumes that people anchor on the previous order quantity and adjust it towards prior demand realisation (Schweitzer and Cachon, 2000).

Although the PTC effect has been proved pervasive and robust, the relative strength of PTC effects is likely to be moderated by task and contextual factors (Moore and Healy, 2008). Discrepancies are inherent either in different countries, different particularities of experiments (e.g., information and feedback, cost profile, market environment, decision complexity), or different features of subject pools (e.g., sex, cognitive reflection). Similar rules apply to overconfidence and insufficient adjustment heuristic. Wright and Phillips (1980) and Lee et al. (1995) have found variations in overconfidence exist in different countries. For mean anchoring, the estimated values of the anchor factors vary widely within [0, 1], where a low anchor factor means a weak anchoring tendency. They can be as low as 0.20 (Bolton et al., 2012, trained graduate students) or as high as 0.79 (Schweitzer and Cachon, 2000, low profit setting). Such disparity has been confirmed to be attributed to different subject pools (Bolton et al., 2012) and cognitive reflection (Moritz et al., 2013). For demand chasing, its anchor factor is estimated to be approximately 0.10 (Bostian et al., 2008; Lurie and Swaminathan, 2009), but censored demand will result in stronger demand chasing and worse performance (Lurie and Swaminathan, 2009), and individuals with higher cognitive reflection exhibit a lower tendency to chase demand (Moritz et al., 2013).

Given most extant studies have only been carried out in a small number of multicultural areas, there has been no detailed investigation of cultural implications on overconfidence and heuristics in monocultural settings. Such geographical coincidence suppresses the essential extension of theories in monocultural countries and leaves the cross-cultural differences unnoticed, however, these possible differences can bring deeper insight. According to Kroeber and Kluckhohn (1952), ‘culture consists of ideals, values and assumptions about life that guide specific behaviours and are widely shared by people’. Allowing for ubiquitous behavioural differences in cross-cultural studies, most existing experimental research in multicultural countries such as the United States and Germany (see Table 1) is not enough to reveal the effect of culture on behaviour. Multicultural countries are environments in which various ethnic groups collaborate and enter into a dialogue with one another without having to sacrifice their particular identities, and that means diverse behaviour patterns at the individual level and possible neutralised patterns at the aggregate level. The monocultural countries are quite the opposite where prominent behavioural patterns still exist on average.

**Table 1:** Summary of behavioural newsvendor experiments.

**References**	**Location**	**Research focus**	**Cultural concerns**	**Examine the cause of the PTC**	
				**Heuristics**	**Overconfidence**
Schweitzer and Cachon (2000)	USA	Decision biases under known demand		√	
Bostian et al. (2008)	USA	Adapative learning model		√	
Kremer et al. (2010)	Germany	Effect of random errors		√	
Benzion et al. (2010)	Israel	Decision biases under known and unknown demand		√	
Rudi and Drake (2014)	Norway	Impact of demand censoring		√	
Käki et al. (2015)	Finland	PTC effect under supply uncertainty		√	
Fügener et al. (2017)	Germany	Capacity planning		√	
Zhao et al. (2016)	China	Effect of censoring on demand		√	
Feng and Zhang (2017)	China	Duopolistic newsvendor		√	
Lee and Siemsen (2017)	USA	Effect of task decomposition		√	
Lee et al. (2018)	USA	Newsvendor problem, procurement auction and chain contracts		√	
Schultz et al. (2018)	USA	Effect of framing on risk preference		√	
Zhao and Zhao (2017)	China	Competing newsvendor under incomplete information		√	
Zhang et al. (2019)	—	Newsvendor decisions in the presence of strategic customers		√	
Becker-Peth and Thonemann (2018)	Germany	Risk preference		√	
Villa and Castañeda (2020)	Colombia	Power and gender		√	
Surti et al. (2020)	—	Prospect theory, risk and feedback		√	
Li et al. (2019)	China	Individual and cultural differences	√	√	
Feng et al. (2011)	China	Cognitive reflection and cultural backgrounds	√	√	
Ren and Croson (2013)	USA	Overconfidence effect		√	√
This paper	China	Cultural differences on heuristics and overconfidence	√	√	√

This study intends to determine the extent to which the culture changes human's behaviours by guiding the underlying cognitive process and how it could possibly influence them. The hypothesis development is as below.

Among monocultural countries, China is the most well-known owing to its rather long history, massive population, and unique culture. The most widely accepted philosophy in China is *Zhong Yong* or *Doctrine of the Mean*, a doctrine of ‘middle way’ thinking or being moderate. Scholars have applied *Zhong Yong* thinking to explain newsvendors' decision making in China (Chiu, 2000; Feng et al., 2011). For example, Chiu (2000) interprets *Zhong Yong* thinking as ‘holistic information processing and avoidance of extremities in implementation planning’. In the newsvendor context, this philosophy promotes the avoidance of extreme options and a preference for the middle ones, which is likely related to mean anchoring behaviour. We, therefore, postulate that Chinese newsvendors keep mean anchoring throughout newsvendor decisions and provide the following hypothesis:

**Hypothesis 1**. Chinese newsvendors will exhibit significant mean anchoring and insignificant de-bias learning.

The Asian and Western cultures also result in different cognitive styles. Westerners tend to engage in context-independent and analytic perceptual processes, whereas Asians tend to engage in context-dependent and holistic perceptual processes (Nisbett et al., 2001). Compared with Americans, Chinese people exhibit stronger demand chasing behaviour (Li et al., 2019). For this reason, we posit that context-independent and holistic perceptual processes lead to persistent demand chasing behaviour with negligible de-bias learning:

**Hypothesis 2**. Chinese newsvendors chase the prior demand without significant de-bias learning.

Another key attribute of China is that it is highly collectivistic, that is, people act in the interests of the group and not necessarily of themselves. As an important dimension of culture, collectivism and individualism play a role in triggering different goals and different levels of social influence. Elliot (1999) pointed out that interdependent self-construals in collectivistic countries are positively related to the adoption of avoidance goals. As a result, behaviours are directed by a negative event or possibility (Elliot et al., 2001). Additionally, values differ between collectivistic and individualistic countries. If people are socialised as collectivistic, they learn to value interdependence and a sense that ‘we are’. They also tend to engage in social comparisons to ensure they conform to group norms (Kawamura, 2012). Additionally, research in social psychology has proven that the ‘mere presence of others’^1^ can trigger social influences. Specifically, arousal by others' presence strengthens dominant responses, which are correct only in easy or well-learned tasks (Myers, 2009). Consequently, collectivistic people who care about the social comparison are necessarily affected but to a different extent, that is, the performance could be facilitated for those who have learned this task well (e.g., students majoring in business with higher degrees) while hindered for the others. All this above can influence both the process of making ordering decisions and making probability judgments in overconfidence testing, probably in different ways. In a word, overconfidence is context-dependent. Even for the same subject, his/her overconfidence level in ordering decisions can significantly deviate from that in overconfidence testing because the collectivistic culture significantly influences their behaviour especially when involved in the comparison. Therefore, when inspecting the causal relationship between overconfidence and newsvendor decision bias, the scenario of overconfidence test is very important which provides the following hypothesis:

**Hypothesis 3**. Chinese newsvendors' decision bias can be explained by overconfidence.

To test the hypotheses, we applied the laboratory experiments for it bridges the gap between analytical models and real business problems. They are advantageous in establishing causality by randomly assigning subjects to handle endogeneity. Most importantly, they can be replicated in different laboratories which are of great importance given most replications' results were smaller in magnitude and not significant although in the original studies most reported results were large in magnitude and statistically significant (Camerer et al., 2016). Our research contributes to the newsvendor experiment literature by offering the cultural reasons for robust heuristics and revealing whether the long-term learning could be helpful to decrease bias and how it could be more effective. Furthermore, the findings refresh the contextual dependence of overconfidence and provide some useful suggestions for the more reliable test and possible methods to weaken overconfidence.

The remaining part of the article proceeds as follows: Section 2 begins by laying out the theoretical dimensions of the research. Section 3 addresses the experimental processing and origins of the subject pool in detail. Section 4 presents the findings of the research on two key themes: insufficient adjustment heuristics and overconfidence. Section 5 discusses the possible explanations for robust heuristics and the insignificant overconfidence effect from different perspectives. We close with some concluding remarks and suggestions for future research in the conclusion section.

## 2. Theoretical Models

### 2.1. The Classic Newsvendor Problem

In the newsvendor problem, a newsvendor chooses an order quantity *q* to fulfil the uncertain demand of a selling period. Let *D* be the random demand with mean μ, a cumulative distribution function (CDF) *F*(·), and a probability density function (PDF) *f*(·). Further, a rational newsvendor has an unbiased forecast of the demand distribution and makes ordering decisions based on *F*(·). The newsvendor purchases each unit for cost *c* and sells each unit at price *p* > *c*. If inventory overstocks, that is, *q* > *D*, each unsold unit can be salvaged for *s*, where *s* ≤ *c*. The profit of a newsvendor is


(1) $$\begin{array}{r} \pi \left(q\right)=\left(p-s\right)\min\left(q,D\right)-\left(c-s\right)q, \end{array}$$

and its expectation is


$$\begin{array}{l} E\left[\pi \left(q\right)\right]=\left(p-c\right)q-\left(p-s\right)\int_{0}^{q}F\left(x\right)dx. \end{array}$$

For a rational decision maker, there is a unique optimal order quantity given by q^*^ = *F*^−1^(η), where $\eta =\frac{p-c}{p-s}$, as a critical fractile, classifies the products by profit condition. In line with Schweitzer and Cachon (2000), we define a high-profit product if η ≥ 1/2 and a low-profit product otherwise. As η represents the profit margin if the product has zero salvage value, for ease of exposition, we use the profit condition and profit margin interchangeably.

### 2.2. The Behavioural Newsvendor Problem

To revisit the PTC effect in China, we focus on three behaviours: mean anchoring, demand chasing, and overconfidence.

Mean anchoring assumes that the decision maker anchors on the mean demand and adjusts towards the optimal order quantity. Following Schweitzer and Cachon (2000), we define the deviation from the mean demand α_*i*_ as $\left(q_{i}-\mu \right)/\left(q^{*}-\mu \right)$ in the high-margin condition and $\left(\mu -q_{i}\right)/\left(\mu -q^{*}\right)$ in the low-margin condition, where *q*_*i*_ is the *i*-th subject's average order quantity. The closer the α_*i*_ is approaching 0, the stronger the mean anchoring tendency the people have.

Another heuristic we consider is demand chasing, in which subjects anchor on the previous order quantity and adjust towards the most recent realised demand. Specifically, for any given *t*, the linear partial adjustment model is given by:


$$\begin{array}{l} q_{t}=q_{t-1}+\beta \left(D_{t-1}-q_{t-1}\right)+\epsilon_{t}, \end{array}$$

where β represents the degree to which subjects move towards the most recent demand compared to their last choice, with β = 1 implying full demand chasing. ϵ_*t*_ are independent identically distributed (i.i.d.) error terms.

To incorporate the evolution of choices towards the optimum, we further consider the learning effect identified by Benzion et al. (2008). Specifically, the adjustment models for the two heuristics are


$$q_{t}=\{\begin{array}{ll} \mu +\alpha_{t}(q^{*}-\mu )+\epsilon_{t}, & \text{mean anchoring},\\ q_{t-1}+\beta_{t}(D_{t-1}-q_{t-1})+\epsilon_{t}, & \text{demand chasing}, \end{array}$$

where ϵ_*t*_ is an i.i.d. normal error term, α_*t*_ = (1+Δ_α_)α_*t*−1_ and β_*t*_ = (1−Δ_β_)β_*t*−1_ reflect the average degree of rationality in the *t*-th round (e.g., α_*t*_ represents the extent to which subjects deviate from the mean demand μ towards the optimum q^*^). Note that Δ_α_ and Δ_β_ are the percentage changes in the insufficient adjustment bias over time. If the ordering bias diminishes owing to learning, Δ_α_ and Δ_β_ should be positive and less than 1, and α_*t*_ would converge to 1 (implying no bias), whereas β_*t*_ would be approaching 0 (implying no demand chasing).

In addition to the above-mentioned heuristics, overconfidence (in particular, overprecision) has been confirmed to be a cause of the PTC effect. Specifically, Ren and Croson (2013) explained the PTC effect by the fact that market demand perceived by decision makers *D*_*O*_(·) is less risky, that is, *D*_*O*_(·) is a mean-preserving but variance-reducing transformation of the actual demand. Specifically, the perceived demand *D*_*O*_(·) is constructed by mixing the true demand and the demand with zero variance (mean demand μ), that is,


$$\begin{array}{l} D_{O}\left(\cdot \right)=\gamma D+\left(1-\gamma \right)\mu , \end{array}$$

where γ ∈ [0, 1] characterises an individual's level of overprecision. A lower γ implies a higher overprecision level. If γ = 1, then the decision maker is unbiased. This model also explains why overconfident people underorder in the high-margin conditions and overorder in the low-margin conditions.

## 3. Materials and Methods

### 3.1. Participants

Participants were 23 students (11 male and 12 female, ages 19-26) recruited by the online announcement in a Chinese university (see details in Table 2). No participants were excluded from analysis because neither of them did not pass attention checks. The sample size was planned to be comparable with Schweitzer and Cachon (2000) and Feng et al. (2011). To incentivize subjects to take the decisions seriously, they were paid in cash based on the profits they earned in the experiment. Every 100 experimental dollars was worth CNY ¥1 and was added to the show-up fee (CNY ¥25). On average, each subject received CNY ¥45.

**Table 2:** Demographics of participants.

**Age (in years)**				**Sex**		**Educational level**		
**Mean**	**Std. Dev**.	**Min**	**Max**	**Male**	**Female**	**Banchelor**	**Master**	**Ph.D**
22.70	1.94	19	26	47.83%	52.17%	47.83%	43.48%	8.69%

### 3.2. Materials

Fifteen questions were selected from Gino and Moore (2007) and Ren and Croson (2013) for the overconfidence test in the first phase. For example, the question “Xiaoping Deng's age at death (in years) and how confident are you that your answer is within 5% of the right answer?" requires the subject to answer a specific number along with the confidence level that their answers are within 5% of the right answer. Note that we revised the first 10 questions for adjustment to Chinese participants. The second phase involves multi-round decisions with instant feedback, which is realised by a computer interface program using the z-Tree system (Fischbacher, 2007). Detailed questions and screenshots of the experiments are provided in the Appendix.

### 3.3. Design and Procedure

Following the extant studies (e.g., Schweitzer and Cachon, 2000; Ren and Croson, 2013), we design our within-subject experiment with a one-factor that varies with the products' profit margin (products in high- and low-margin conditions). For the sake of comparison, we follow the parameters settings of Ren and Croson (2013): the unit cost of the product *c* = 2 experimental dollars for the high-margin product and *c* = 4 for the low-margin product, and the selling price *p* = 5 experimental dollars and unit salvage value *s* = 1 experimental dollars for both products. The demand is assumed to follow a uniform distribution with *D* ~ *U*[0, 100]. Under these settings, the normative order quantities for high-margin and low-margin products are $q_{H}^{*}=75$ and $q_{L}^{*}=25$, respectively.

The study had two phases: First, subjects were required to answer 15 questions on general knowledge in the form of point estimation and the corresponding confidence level. Their overconfidence levels are their own actual correctness rate divided by their average subjective correctness level. Subjects of overconfidence level less than 1, that is, γ ≤ 1, were classified as overconfident subjects; the lower the level, the more overconfident they were. Then, they had to make 30 rounds ordering decisions in two treatments. For each round, results including the realised demand, overage loss, underage loss, profit, and total profit were shown after inputting the order quantity to ensure that subjects received instant feedback. The whole process of the experiment was conducted in the laboratory and supervised by facilitators. Communication was restricted. After completing the above two phases, they were directed to leave the laboratory and received their cash reward privately. The parameter settings and experimental results are summarised in Table 3.

**Table 3:** Parameter settings and characteristics of participants.

**Treatment**	**Parameters**	**No. of Participants**	**Average orders**	**Average overconfidence level**
High profit (HM)	price = 5, cost = 2, salvage = 1 $q_{H}^{*}=75$	23	45.12^***^ [13.03]	0.52 [0.28]
Low profit (LM)	price = 5, cost = 4, salvage = 1 $q_{L}^{*}=25$	23	35.56^***^ [10.75]	

*Standard deviation in brackets. t-test vs. optimal*.

*** *p < 0.01*.

### 3.4. Analysis Plan

We take the following approach to analyses: all the subjects' data including their overconfidence testing results, the 30 rounds ordering decisions, and the 30 rounds realised demand results are kept because the experiment is strictly under control. The analysis of mean anchoring behaviour involved *t*-test and partial-adjustment model with autoregressive dynamics identified by Bostian et al. (2008). We calculate the average α_*i*_, denoted by $\bar{\alpha }$, in the high- and low-margin cases, compare them with zero deviation ($\bar{\alpha }=1$), and examine whether there is a significant difference. After making anchoring factor to be time-varying, i.e., α_*t*_ = (1+Δ_α_)α_*t*−1_, we apply the partial-adjustment model, i.e., $q_{t}=\mu +\alpha_{t}\left(q^{*}-\mu \right)+\epsilon_{t}$, to detect learning effect by Δ_α_. Correspondingly, demand chasing behaviours are captured by the partial-adjustment model, i.e., *q*_*t*_ = *q*_*t*−1_ + β(*D*_*t*−1_ − *q*_*t*−1_) + ϵ_*t*_, and the learning effect is detected by Δ_β_ when β is time-varying, i.e., β_*t*_ = (1−Δ_β_)β_*t*−1_.

We use the random effect model to examine the overconfidence effect, where a total of 1,380 observations of panel data is used. Following Ren and Croson (2013), we used the difference between the *i*-th individual's decision in the *t*-th round *q*_*it*_ and the optimal order $q_{j}^{*}$ in the *j* treatment as the dependent variable, referred to as the individual error. We first regress individual error against overconfidence level (main independent variable) to verify the negative linear relationship between them (Ren et al., 2017). Further, we examine the interaction between overconfidence effect and demand chasing behaviour, including two other independent variables^2^, prior order anchoring, i.e., *q*_*t*−1_ and insufficient adjustment derived by the difference between the prior demand and prior order, i.e., *D*_*t*−1_ − *q*_*t*−1_. Specifically, the regression equation is


$$\begin{array}{l} I_{j}\left(q_{it}-q_{j}^{*}\right)=\theta +\Sigma \lambda_{i}X_{i}+\varepsilon_{i}+\epsilon_{it}, \end{array}$$

where *i* = 1, 2, ⋯ , 30, *j* = *L, H*. {*X*_*i*_} is the set of the above independent variables, λ_*i*_ is the corresponding parameters, and ε_*i*_ captures the unobserved individual heterogeneity. Note that *I*_*j*_ equals 1 in the low-profit (L) condition and –1 in the high-profit (H) condition.

## 4. Results

In this section, we present the general results of our experiment as well as revisions to heuristics (including mean anchoring and demand chasing) and the overconfidence effect. Moreover, we summarise our results for response to the hypotheses.

### 4.1. General Results

**Figure 1:**
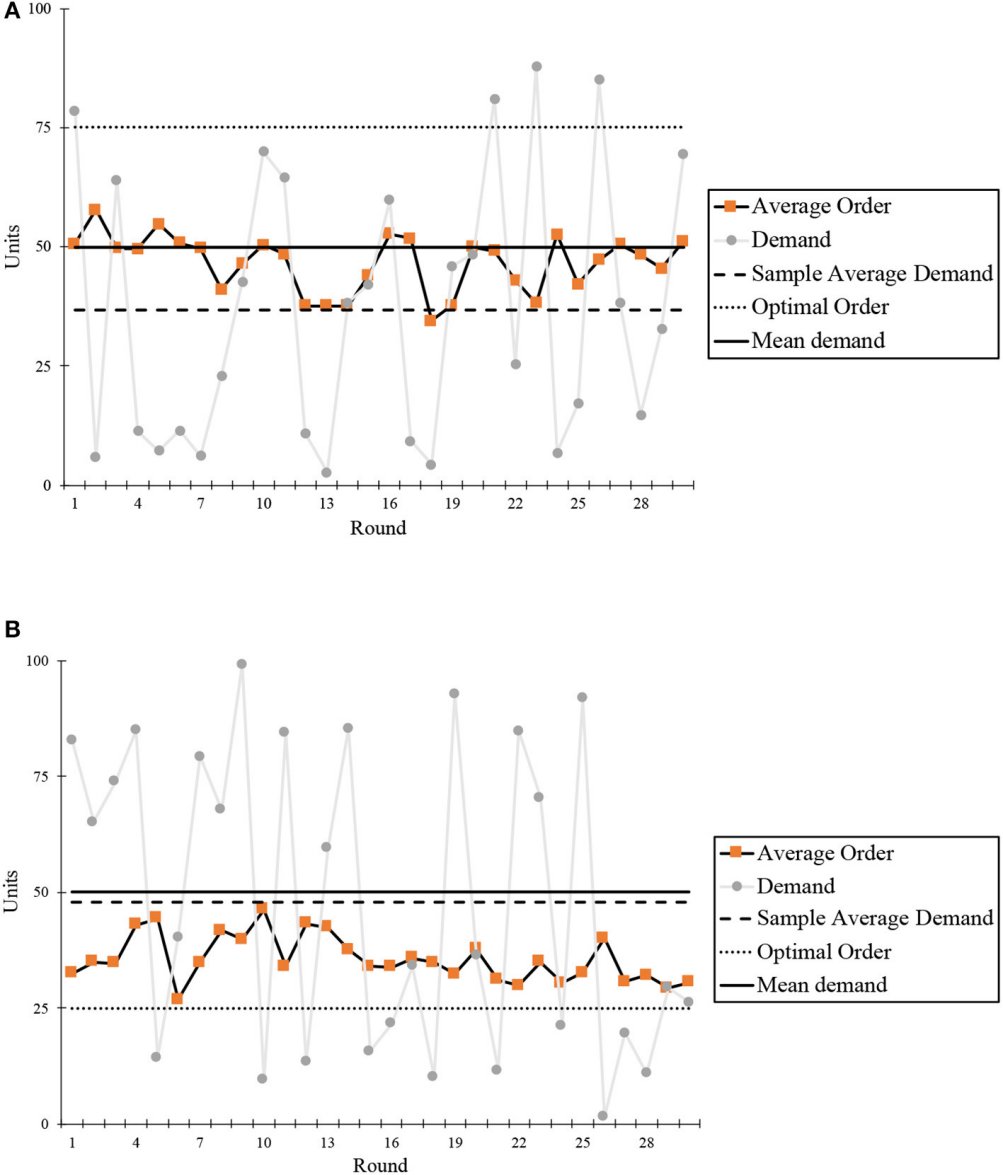
Ordering quantity per round. **(A)** High margin and **(B)** Low margin.

As Figure 1 shows, our experimental results did not show a perfect PTC effect, for average ordering is less than the mean demand in the high margin. Nevertheless, we cannot say the robustness of the PTC effect is challenged because the PTC effect still exists among Chinese subjects after revisiting the centre to the SAD.

### 4.2. Heuristics and Insufficient Adjustment Revisited

Table 4 summarises the regression results of our experiment by separately regressing the data in the high-margin and low-margin conditions. We examine the mean anchoring behaviour by applying the *t*-test to compare the $\bar{\alpha }$ against 1. If $\bar{\alpha }$ is significantly lower than 1, subjects show the mean anchoring behaviour. Further, we consider the learning effect of mean anchoring by letting α be time-varied (α_*t*_) and estimate Δ_α_ to test whether there is significant de-bias learning. Similarly, the demand chasing behaviour and its learning effect are also examined in both treatments.

**Table 4:** Regression results.

	**High margin**	**Low margin**
Mean anchoring		
$\bar{\alpha }$	−0.13^***^(0.000)	0.58^***^(0.000)
Mean anchoring with learning		
α_1_	−0.10(0.210)	0.44^***^(0.000)
Δ_α_	0.02(0.592)	0.02^**^(0.012)
α_30_	-0.18	0.74
Demand chasing		
β	0.11^**^(0.015)	0.12^***^(0.002)
Demand chasing with learning		
β_1_	0.07(0.287)	0.13^**^(0.031)
Δ_β_	−0.02(0.628)	0.02(0.576)
β_30_	0.12	0.08
Overconfidence	−12.95(0.139)	4.65(0.675)

*p-value in brackets*.

** *p < 0.05*

*** *p < 0.01*.

**Figure 2:**
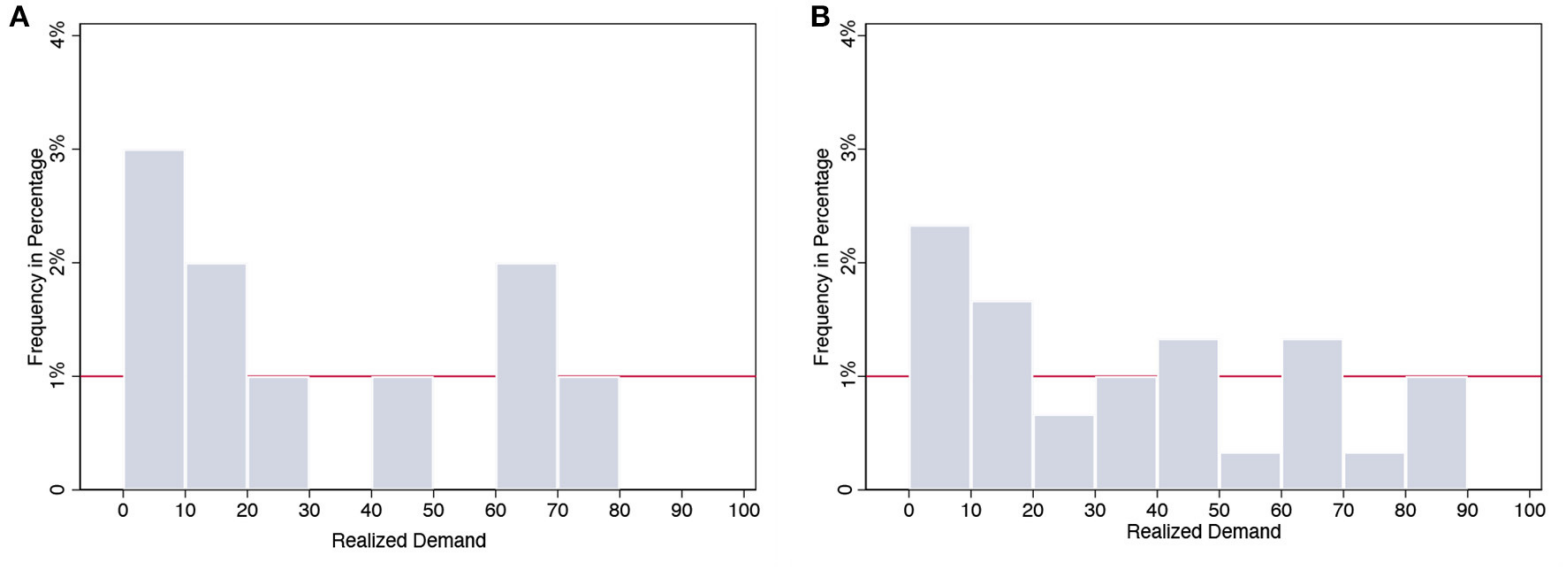
Histogram of realised demand. **(A)** High margin (first 10 rounds) and **(B)** High margin (30 rounds).

Chinese participants deviated their ordering quantity from optimum to mean demand (50) significantly ($\bar{\alpha }=-0.13$, 95% confidence interval is [−0.311, 0.048] in the high-margin condition and $\bar{\alpha }=0.58$, 95% confidence interval is [0.388, 0.768] in the low-margin condition). Regarding the de-bias learning effect, participants did not show significant learning and adjustment whenever anchoring on mean demand (Δ_α_ = 0.02, *p* = 0.592) or anchoring on SAD=36.9 (Δ_α_ = −0.02, *p* = 0.189) in high-margin conditions, where there is a large discrepancy between mean demand and SAD (see histogram in Figure 2). On the contrary, disparity is narrow in low-margin condition, in which they can improve their performance by overcoming insufficient adjustment bias round by round (Δ_α_ = 0.02 is positive, *p* = 0.012, and α_30_ = 0.74 is converging to 1).

Demand chasing, as a well-known heuristic to explain the decision bias, is strongly supported in our Chinese newsvendor experiment. The estimated parameter β was 0.11 (*p* = 0.015) in high-margin condition and 0.12 (*p* = 0.002) in low-margin condition, and demand chasing behaviour persists throughout the experiment because the parameter Δ_β_ is insignificant in both conditions (Δ_β_ = −0.02, *p* = 0.628 in high-margin condition and Δ_β_ = 0.02, *p* = 0.576 in high-margin condition).

### 4.3. Overconfidence Revisited

Linear regression results reveal that overconfidence is invalid (*p* = 0.139 in the high-margin condition and *p* = 0.675 in the low-margin condition) in explaining the ordering bias. Considering the robust demand chasing behaviour, possible interference should be incorporated when inspecting the robustness of the overconfidence effect. Table 5 summarises further multiple regression results. We find an insignificant overconfidence effect (*p* = 0.578) on the level of individual errors [column (1)]. Columns (2)–(4) further test the interference of demand chasing behaviour by further including *prior order anchoring* and *insufficient adjustment*, respectively, in columns (2)–(3), and together in column (4). Results reaffirm the robustness of demand chasing and insignificance of the overconfidence effect (*p* = 0.428, 0.501, 0.431, respectively).

**Table 5:** Multiple regression results for overconfidence and demand chasing (random effects).

	**(1)**	**(2)**	**(3)**	**(4)**
Overconfidence	–4.15	–5.84	–4.95	–5.79
	(0.578)	(0.428)	(0.501)	(0.431)
Prior order anchoring		0.17^***^		0.13^***^
		(0.000)		(0.000)
Insufficient adjustment			–0.07^***^	–0.04^**^
			(0.000)	(0.017)
Constant	20.45^***^	13.95^***^	20.84^***^	15.80^***^
	(0.000)	(0.000)	(0.000)	(0.000)
*R* ^2^	0.016	0.034	0.025	0.038

*p-value in brackets*.

** *p < 0.05*

*** *p < 0.01*.

### 4.4. Summary

In summary, the mean anchoring behaviour is as strong as predicted by Zhong Yong but anchoring on the mean demand is not a certain option for Chinese in the long term. De- bias learning effect can be significant if realised demand is highly consistent with theoretical demand results, which means the narrower cognitive gap between fact and theory can help the subjects better understand the questions and adjust to the optimum. Hypothesis 1 is supported.

Results also confirm Hypothesis 2 that demand chasing behaviours are significant even in the long run. Furthermore, adjustment to the optimum is not recognisable within 30 rounds in contrast to mean anchoring. The insignificant learning process suggests that the underlying context-dependent and holistic perceptual processes are hard to overcome in a short time.

Furthermore, the multiple linear regression results reveal that the overconfidence effect remains insignificant, even when allowing for the influence of demand chasing. Overconfidence fails to explain the decision bias. Hypothesis 3 is not supported.

## 5. Discussion

Examining heuristics and overconfidence under the monocultural background have given insight into whether the culture changes human behaviours or not. Results of mean anchoring and demand chasing behaviours have successfully supported the cultural implication on behaviours. The invalid overconfidence effect offers an opportunity to deeply inspect the nature of overconfidence, especially under the collectivistic culture. In this section, we first discuss cultural reasons robust heuristics and different learning performances. Second, we offer some possible explanations for the unfunctional overconfidence effect through the lens of a gap between judgement and decision: motivation, cultural implications, controllability, and complexity. Last, we offer our thoughts on which centre works in the PTC effect when SAD deviates from mean demand.

### 5.1. Why Heuristics Are Robust

*Zhong Yong* philosophy in China is an obvious cultural cause of mean anchoring behaviour because Chinese people feel safe to place orders based on the median demand. But how much power it has in influencing their behaviours remains a mystery—the mean anchoring behaviour can be weakened by long-term learning. The narrower gap between the distribution of realised demand and theoretical distribution can result in a shorter learning process, which implies mean demand can be an available option at the beginning but this safety-seeking strategy can be easily broken under uncertainty.

The individual cognitive style plausibly explains the demand chasing behaviour of Chinese subjects. As mentioned in the introduction, in contrast to Westerners, the Chinese tend to engage in context-dependent and holistic perceptual processes. The fallacy of context-dependence makes the previous demand an important part of the whole picture and influences the present decision. Results also show that demand chasing is a strong heuristic for people not to extricate from. Even a longer learning process cannot necessarily dictate less bias because cognitive style not only confuses the concept of independent randomness but also influences the underlying thinking, learning, and information processing processes.

### 5.2. Why Overconfidence Cannot Explain Decision Bias

The insignificant overconfidence effect indicates that probability judgement (overconfidence testing) and newsvendor decisions are possibly distinct for Chinese subjects. As a result, the unconnected relationship between them leads to the unpredictability of the overconfidence level for ordering bias.

The gap lies first in motivation. When they make probability judgements in the overconfidence pre-testing, there is no motivation or punishment. However, outcomes of ordering decisions in the newsvendor setting are directly connected to subjects' earnings. As Heckhausen and Heckhausen (2008) wrote, ‘explicit motives are the core of action control which provide directionality of behaviour and a criterion for success’, the accuracy of the confidence level heavily depends on the explicit motives to ensure the same devotion to overconfidence testing and decision making. Without these motives comes a mismatch of cognitive processes between judgement and decision making and inconsistent overconfidence levels.

The second difference is rooted in the collectivistic culture. Regarding people in collectivistic cultures, Markus and Kitayama (1991) said that they ‘see themselves as connected to others, define themselves in terms of relationships with others, and see their characteristics as more likely to change across different contexts’. The cultivation of collectivistic individuals starts in the early stages of socialisation. Childrens' activities include a large amount of time spent with the extended family, unpaid childcare for younger siblings and cousins, and hard work in school to bring honour to the family (Baldwin et al., 2006). On this account, individuals who ascribe to collectivistic values also tend to engage in social comparisons to ensure they conform to group norms. Consequently, people always enter into social competition and seek self-satisfaction from excelling, especially in academic skills and results (He et al., 2021). While overconfidence testing cannot induce social comparison for no visible bonuses, performance in ordering decisions is directly related to financial rewards, which can easily make people more cautious about living up to social expectations. Although information about personal financial rewards was confidential throughout the whole experiment, the ‘mere presence of others’ can trigger social influences and result in facilitated performance for those who have learned this task well (e.g., students majoring in business with higher degrees) while opposite for the others. Therefore, there are diverse changes in their overconfidence level and performance; this disturbs the regression results of the overconfidence effect.

The ordering decisions are unlike judgements in overconfidence testing in terms of controllability as well. In the test, they can lower the confidence level to match their uncertain estimation, that is, the probability judgement on accuracy and estimation is interactive, and both of them are controllable. By contrast, the ordering decision is based on stochastic demand, which is out of their control. As a result, different psychological states can lead to incompatible levels of confidence.

We can also interpret this difference through the complexity of the decision making process as it is compounded by many judgements. According to Aronson and Aronson (2018), ‘Most decisions involve a two-step process’. Humans' automatic system first produces a quick-and-dirty assessment of reality, an intuition and unthinking preference, just like the first forecast about demand in newsvendors. Then, people use more controlled or deliberate thinking to modify the initial impressions after motivation or further information. As a result, financial rewards and historical records in the ordering decision can arouse the second stage of thinking, leading to the unpredictable link between overconfidence level in the test and decision making.

**Figure 3:**
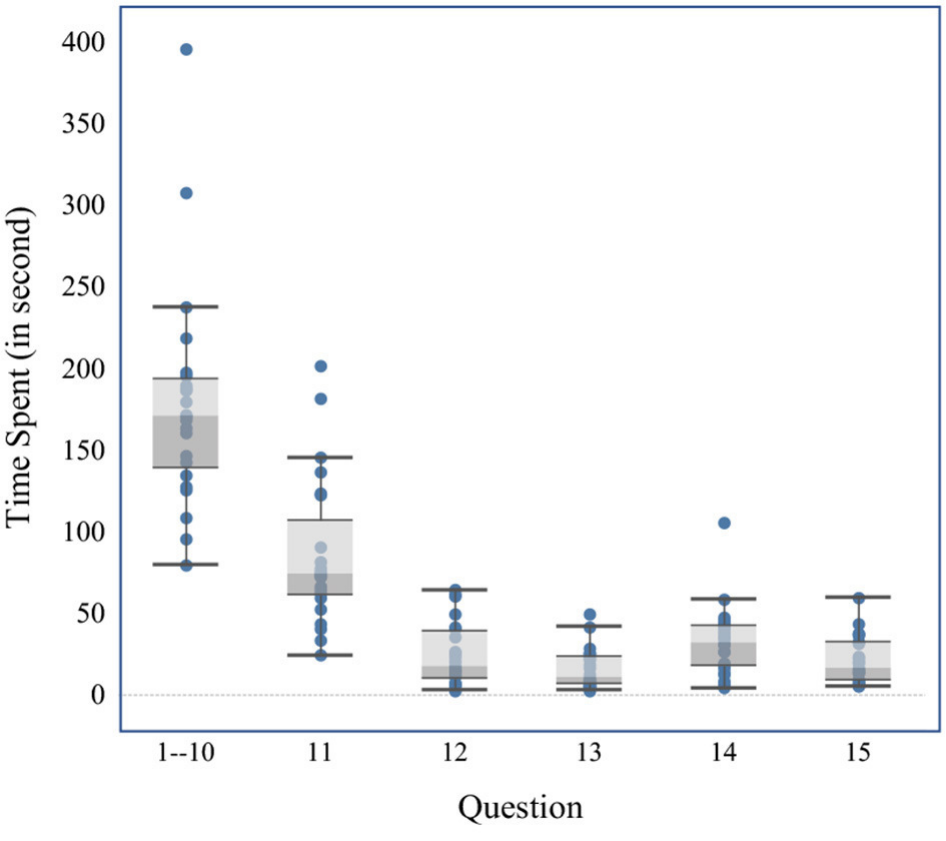
Time spent on confidence level test. Time spent on 1–10 questions is on average.

In a word, doubts about the testing measures of overconfidence cannot be erased. Numerous studies have implicated the ‘extensiveness’ or ‘thoroughness’ of people's judgement, and overconfidence tends to diminish when people think about problems more extensively (Sieck and Yates, 2001; Soll and Klayman, 2004). However, the repeated questions about general knowledge that involves guessing, will bore subjects and lead to distraction (Figure 3 shows that subjects take less time to think round by round). Besides, different types of overconfidence testing, that is perceptual tasks or cognitive tasks, also result in miscalibration (Stankov et al., 2012; Burns et al., 2016). Both task characteristics and cultural backgrounds should be incorporated when refining the test.

### 5.3. Why Centre Needs Re-Definition

There are several causes that subjects' centre deviate from mean demand. Interdependent self-construal in collectivistic culture is positively related to the adoption of avoidance goals (Elliot, 1999); that is, the behaviour is instigated or directed by a negative event or possibility in avoidance motivation. In the first 20 rounds in our experiment, there was extremely low demand (3 units), incurring great losses among subjects. For avoidance purposes, the subjects tended to be more conservative, which means less ordering quantity; therefore, the average order quantity can be less than the mean demand. Besides, demand chasing behaviour can also lead to the centre deviating from mean demand. Demand chasing individuals would place their order based on realised demand therefore average ordering quantity is close to SAD. As a result, it is the not mean demand but the sample average demand (SAD) that decides the PTC centre which means the PTC effect is a robust phenomenon with dynamic psychological constructs.

## 6. Conclusion

In this study, inspired by the notable findings in cross-cultural research, we re-examined the PTC effect and its plausible causes through experiments in mainland China, trying to reveal the cultural implications on human behaviours. Our experiment confirmed that the PTC effect exists for Chinese subjects, but the centre needs revisiting to the average of the realised demand which determines the boundary of the PTC effect. This implies that in a real situation, people perceive the demand distribution as dynamic and continually adjust their judgement about the mean demand based on average realised demand. We also confirmed that demand chasing and mean anchoring behaviours are significant, and it is difficult for subjects to make decisions without heuristics. Nevertheless, the de-bias learning processes are different for these two heuristics. Mean anchoring behaviour can be weakened through learning but only under the condition when realised demand are highly conformed to theoretical distribution. However, demand chasing is more robust in China, as it is consistent throughout the whole experiment without a recognisable learning effect.

The most interesting finding from this study is that the overconfidence effect is not always valid in explaining the PTC effect, especially for Chinese newsvendors. This could be attributed to the mismatch in judgement and decision derived from the motivation gap, collectivistic culture, and differences in controllability and complexity. Specifically, financial motivation as an explicit motive is the core of action control and can encourage individuals to concentrate, whereas, without such a motive, people can become distracted, leading to inconsistent confidence levels. Collectivistic individuals are highly involved in social comparison, and this pressure can strengthen performance for well-learned people and hinder others. That implies personal traits are important controlled variables to explain the overconfidence effect. Overconfidence of people in the collectivistic society can be moderated by their capability, which offers some possibility to manipulate the overconfidence by reminding them of their ignorance.

This paper adds to the growing body of research that people with different cultural backgrounds behave differently and calls for more exploration of overconfidence, as a complex mechanism. We raised an important question concerning the nature of overconfidence. Whether it is a robust explanatory variable independent of other factors, or it can be moderated by different factors in different cultural backgrounds? Our work is closely related to Li et al. (2019) and Feng et al. (2011), who studied the cross-national differences in operations decisions. While they mainly studied heuristics, we complemented their results by also considering the overconfidence effect. Furthermore, we found that the ‘centre’ should be re-defined as the SAD to retain the robustness of PTC.

Increasing the sample size could lend additional support to our claims and perhaps lead to a deeper inspection of the gap between judgement and decision. Despite its exploratory nature, this study offers some insights into the cultural effect on judgement and decision making processes. Future research can conduct large randomised controlled trials to test the possible interaction between personal traits, cultural backgrounds, and overconfidence effect. Although a statistically significant overconfidence effect is not found in our experiment, we still cannot deny the explanatory power of overconfidence in Chinese newsvendors' ordering bias. We could further verify our conclusion by improving the experiment in the following aspects: first, the cultural background should be considered in the overconfidence testing. The properties of subjects may not influence the general conclusion in a multicultural society because diversity eliminates individual differences, whereas, in monocultural countries, the impact of culture on decision makers cannot be ignored. For example, the social comparison must be controlled in a collectivistic culture, possibly through separate online experiments or asynchronous processes to inhibit in-process comparison. Task dependence should also be considered. Sufficient training before making decisions is crucial to narrowing the difficulty gap between overconfidence testing and decision making. However, the extent to which people should learn about newsvendors' decisions to achieve similar confidence levels remain unclear. In addition to improvement of point estimation, various measures to test overconfidence (e.g., interval estimation or half-range format, behavioural measures) can be assessed for alternatives, which may provide data of higher quality and reliability.

## Data Availability Statement

The raw data supporting the conclusions of this article will be made available by the authors, without undue reservation.

## Ethics Statement

The studies involving human participants were reviewed and approved by the Research Ethics Committee of Business School, Sichuan University. The patients/participants provided their written informed consent to participate in this study.

## Author Contributions

LY analysed the research and wrote the manuscript. DC designed and performed the research. Both authors contributed to manuscript revision, read, and approved the submitted version.

## Funding

The present study was sponsored by Sichuan University.

## Conflict of Interest

The authors declare that the research was conducted in the absence of any commercial or financial relationships that could be construed as a potential conflict of interest.

## Publisher's Note

All claims expressed in this article are solely those of the authors and do not necessarily represent those of their affiliated organizations, or those of the publisher, the editors and the reviewers. Any product that may be evaluated in this article, or claim that may be made by its manufacturer, is not guaranteed or endorsed by the publisher.

## Footnotes

^1^‘Mere presence’ means people are not competing, do not reward or punish, and in fact do nothing except be present as a passive audience or as co-actors.

^2^According to Kirshner and Moritz (2020), the regression model on demand chasing can be extended to a partial adjustment model in which subjects partially anchors on the prior period order.
