# Supplementary material for: Revisiting the Causes of the Pull-to-Centre Effect: Evidence From China
Source: Front Psychol. 2022 Feb 2;12:754626. doi: 10.3389/fpsyg.2021.754626 (PMC8847743; doi:10.3389/fpsyg.2021.754626)
Supplement: Supplementary file 1 [file Data_Sheet_1.zip › Supplementary Material Presentation/decision.pdf]

Welcome to Our Experiements

During this experiment you will act as a manager, who makes ordering decisions for a retailer. The experiment will take about an hour.

Instructions

Your earnings from the session today will be determined by your decisions in this task. In this task you will earn profits in experimental dollars. At the end of the session each 100 experimental dollars will be worth 1 Chinese Yuan (RMB).

In this task, you will act as a seller of a product. In each round, you need to decide how much of this product to order from a supplier. You will then have the opportunity to sell your product to consumers.

Each unit you order costs 2.0 experimental dollars. Each unit you sell earns 5.0 experimental dollars. The number of units you sell will depend on market demand and how much you ordered. At the end of each round, you will return any unsold units to your supplier and get back 1.0 experimental dollars per unsold unit (salvage value). Units cannot be saved for following rounds.

You will be making this decision for 30 rounds.

CONTINUE

Instruction (cont.)

Market Demand

In each round, the computer will randomly select a market demand quantity from a known distribution which will be described later.

The market demand in any round is independent of the market demand in previous or later rounds. Thus, if demand is small (or large) today, this will not affect whether demand is small (or large) tomorrow.

Profit

If market demand is greater than the amount you ordered, then you will sell the number of units you ordered. If market demand is smaller than the amount you ordered, then you will sell the number of units demanded and return the rest to your supplier.

Your profits for this session will be the sum of your profits in each round. Your profit in each round is the number of units you sell times 5.0 experimental dollars each, minus the number of units you purchased times 2.0 experimental dollars each, plus the number of unsold units you returns to your supplier times 1.0 experimental dollars each.

Your profits equal: Quantity sold X 5.0- Quantity ordered X 2.0 + Unsold quantity X 1.0

After you place an order, you will be told the market demand and your resulting profit. You will also see a table of your past decisions and resulting profits.

READY

Period

1 of 30

Remaining time [sec]: 18

Market Information:

Selling Price: 5.0
Buying Cost: 2.0
Salvage Value for Unsold Units: 1.0

Enter Your Order Quantity:

Place Order

Please use only whole numbers.

The market demand will be randomly drawn from a uniform distribution with a lower bound 0, a upper bound 100 and a mean (average) of 50.

Period

1 of 30

Remaining time [sec]: 45

Results

Selling Price: 5.0
Buying Cost: 2.0
Salvage Value for Unsold Units: 1.0

Quantity Ordered: 22 **Actual Demand:** 73
Quantity Sold: 22 **Unsold Quantity:** 0
Profit in this round: 66.0

Round	Actual Demand	Unsold Quantity	Overage Loss	Shortage	Underage Loss	Order Quantity	Profit	Total Profit
1	73	0	0	51	152	22	66.0	67.0

Please press Next to begin the next round: NEXT
