# Supplementary material for: Revisiting the Causes of the Pull-to-Centre Effect: Evidence From China
Source: Front Psychol. 2022 Feb 2;12:754626. doi: 10.3389/fpsyg.2021.754626 (PMC8847743; doi:10.3389/fpsyg.2021.754626)
Supplement: Supplementary file 1 [file Data_Sheet_1.zip › Supplementary Material Presentation/over.pdf]

Please provide your best estimate of the answers to these questions:

	Estimation	The probability that your answer is right
1. Xiaoping Deng's age at death (in years)	22	0 ○ ○ ○ ○ ○ ○ ○ ○ ○ ○ ○ 100%
2. Mr. Barack Obama's age at which he was elected to the U.S. President)	22	0 ○ ○ ○ ○ ○ ○ ○ ○ ○ ○ ○ 100%
3. What is the playing time of the national anthem of China? (in seconds)	22	0 ○ ○ ○ ○ ○ ○ ○ ○ ○ ○ ○ 100%
4. Number of books written by Louis Cha (Jin Yong)	22	0 ○ ○ ○ ○ ○ ○ ○ ○ ○ ○ ○ 100%
5. What is the boiling point of water at the top of Mount Everest (in Celsius)	22	0 ○ ○ ○ ○ ○ ○ ○ ○ ○ ○ ○ 100%
6. Height of a newborn giraffe? (in centimeters)	22	0 ○ ○ ○ ○ ○ ○ ○ ○ ○ ○ ○ 100%
7. Year in which First Lady Melania Trump was born (A.D.)	22	0 ○ ○ ○ ○ ○ ○ ○ ○ ○ ○ ○ 100%
8. Number of red stripes on the national flag of the USA	22	0 ○ ○ ○ ○ ○ ○ ○ ○ ○ ○ ○ 100%
9. Air distance from Harbin to Urumqi (in kilometers)	22	0 ○ ○ ○ ○ ○ ○ ○ ○ ○ ○ ○ 100%
10. Height of the shortest basketball player in NBA (in centimeters)	22	0 ○ ○ ○ ○ ○ ○ ○ ○ ○ ○ ○ 100%

Please press Continue when you are finished CONTINUE

Suppose you are planning to participate in a lottery game. Each day there is a 60% chance you will win $1 and a 40% chance that you will lose $1. Below are the paths that 10 other people's lotteries have taken in the first 150 days of playing this game.

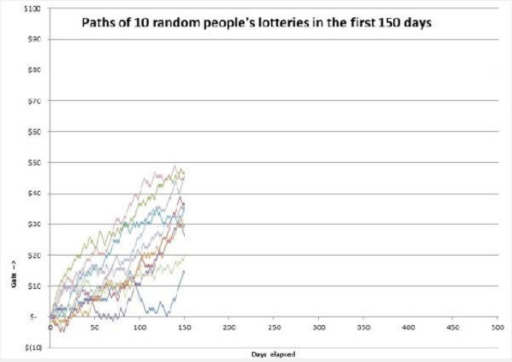

You are about to play. Please use your intuition to estimate the likely outcome for the change in your wealth after 500 days.

How much money will you end up with after 500 days?

How confident are you that your answer is within 5% of the right answer? 0 ○ ○ ○ ○ ○ ○ ○ ○ ○ ○ ○ 100%

CONTINUE

If we asked you to estimate how old the person pictured above is, you probably wouldn't be exactly sure.

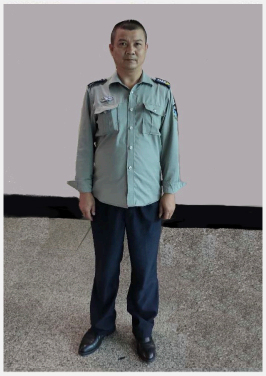

Please try your best and give us your best estimate.

How old is the person in the picture above? (enter the number of years)

How confident are you that your answer is within 5% of the right answer? 0 ○ ○ ○ ○ ○ ○ ○ ○ ○ ○ ○ 100%

CONTINUE

If we asked you to estimate how much the person pictured above weighs, you probably wouldn't be exactly sure.

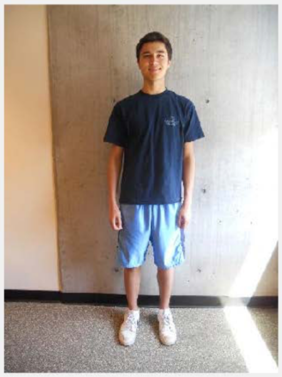

Please try your best and give us your best estimate.

Enter your estimate of the person's weight in kilos.

How confident are you that your answer is within 5% of the right answer? 0 ○ ○ ○ ○ ○ ○ ○ ○ ○ ○ ○ 100%

CONTINUE

Suppose a jumping bean is lying on a sloped sidewalk. Each jump has a 75% chance of moving the bean 1 inch to the left and a 25% chance of moving the bean 1 inch to the right. Please use your INTUITION to answer the following.

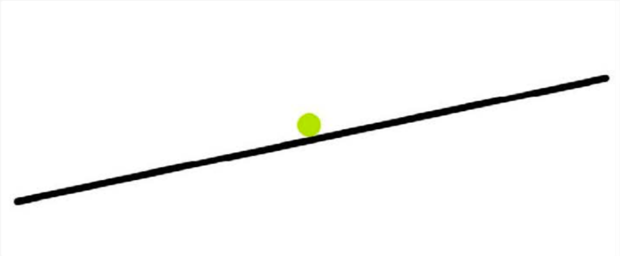

How many inches away from its starting point will the jumping bean be after 600 jumps?

To the left/right of where it started ○ Left ○ Right

How confident are you that your answer is within 5% of the right answer? 0 ○ ○ ○ ○ ○ ○ ○ ○ ○ ○ ○ 100%

CONTINUE

Suppose you had invested $100 in the stock of Tesla Inc. on Oct 31, 2013 and you didn't sell any stock. What is your guess about how much that investment would have been worth on October 31, 2019?

Without looking up historical data, please use your intuition to estimate the possible value.

What dollar amount do you think the investment would have been worth on October 31, 2019?

How confident are you that your answer is within 5% of the right answer? 0 ○ ○ ○ ○ ○ ○ ○ ○ ○ ○ ○ 100%

CONTINUE
